# JOB AVAILABILITY FOR OLDER WORKERS IN A COMMUNITY: ANALYSIS ON JOB POSTINGS

**DOI:** 10.1093/geroni/igad104.2394

**Published:** 2023-12-21

**Authors:** Hyesu Yeo

**Affiliations:** University of Georgia, Athens, Georgia, United States

## Abstract

**Objectives:**

This study examined the current job availability in a local job market for older workers and investigated the required skills the older workers to be competitive.

**Background:**

Most studies approached older workers’ employment transition from the supply side of the labor market, examining individual-level factors for a successful employment transition. However, there is a lack of knowledge on the demand side, such as job opportunities, regarding older workers’ employment transition.

**Methods:**

The study uses combined data with job postings collected in the 4th quarter of 2021 from three engines and the O*NET database with occupational information to identify job opportunities and required skills. Additionally, the 2020 and 2021 American Community Survey were used to explore the characteristics of the older workforce in the community.

**Results:**

A total of 5,160 job postings divided into 468 occupations were found in the community. The most frequently available jobs were healthcare (14%), transportation and material moving (13 %), and sales (13%) occupations, which required some work experience needed. The older labor force mostly had a college or associate degree (45.8%) and was in educational (16%), office (11%), and sales (11%) occupations. All occupations require technological skills.

**Conclusion:**

The study found a mismatch between the available jobs and the actual older labor force, and those who are in employment transition, even downward movement, would be required some related experience. These suggest that a pre-employment transition program providing job training and age-friendly employment support services would be helpful for older workers in all occupational classes.

